# Identification and validation of aging-related genes in patients with multiple myeloma

**DOI:** 10.3892/ol.2026.15739

**Published:** 2026-07-02

**Authors:** Huajing Liu, Senhua Song, Yingtao Wu, Yu Wang, Yueping Liu, Ying Liu

**Affiliations:** 1 Department of Laboratory Medicine, General Hospital of Central Theater Command, Wuhan, Hubei 430000, P.R. China; 2 Post-Doctoral Research Center, General Hospital of Central Theater Command, Wuhan, Hubei 430000, P.R. China; 3 Department of Hematology, General Hospital of Central Theater Command, Wuhan, Hubei 430000, P.R. China; 4 Hubei Key Laboratory of Central Nervous System Tumor and Intervention, Wuhan, Hubei 430000, P.R. China; 5 Department of Disease Prevention and Control, General Hospital of Central Theater Command, Wuhan, Hubei 430000, P.R. China

**Keywords:** multiple myeloma, bioinformatics, aging-related genes, TXN

## Abstract

This study aimed to identify and validate aging-related genes (ARGs) implicated in multiple myeloma (MM), thereby advancing the understanding of the molecular mechanisms underlying the disease. mRNA expression data were retrieved from the Gene Expression Omnibus, and used as a training set to identify ARGs. Gene Ontology and Kyoto Encyclopedia of Genes and Genomes enrichment analyses were conducted to explore the functional roles of the ARGs in MM. Candidate genes identified through least absolute shrinkage and selection operator (LASSO) logistic regression and support vector machine recursive feature elimination (SVM-RFE) were compared using a Venn diagram, which revealed the overlap between the genes identified by the two algorithms. A receiver operating characteristic curve was generated based on the screening results. The candidate genes were further validated using the GSE39754 and GSE5900 datasets. Real-time quantitative polymerase chain reaction (RT-qPCR) was employed to validate the mRNA expression of key genes. Compared to normal individuals, patients with MM exhibited differential expression of 19 genes, of which 5 were upregulated and 14 downregulated. A total of 6 candidate genes (TXN, JUN, FOS, HIF1A, CAT and KCNA3) were identified through LASSO regression and SVM-RFE screening. Among them, TXN exhibited the most significant differential expression, suggesting its potential as a diagnostic biomarker for MM. In addition, *in vitro* RT-qPCR analysis confirmed that the mRNA levels of TXN in MM cells aligned with the bioinformatics findings, showing higher expression compared to normal B-lymphocyte cell lines. In conclusion, this study identified age-associated molecular patterns in MM and highlighted the diagnostic potential of TXN, offering novel insights for clinical applications in MM.

## Introduction

Multiple myeloma (MM) is a malignant hematologic tumor that originates in the bone marrow. Annually, ~588,161 individuals worldwide are diagnosed with MM (1). In 2020, among 1,278,362 cases of leukemia, lymphoma and MM, 176,404 cases of MM were reported, accounting for 14% of the total (2). Despite the unprecedented response and survival rates in MM treatment, the disease remains considered incurable due to its complex pathogenesis, recurrence and drug resistance (3). Furthermore, MM manifests with various clinical symptoms, leading to misdiagnosis and underdiagnosis, which can delay optimal treatment and pose a significant threat to patients' lives (4). Therefore, it is crucial to explore the pathogenesis of MM and identify reliable laboratory diagnostic biomarkers.

Aging is a natural and inevitable process that occurs over time in organisms (5). Pathologically, aging results from a cumulative accumulation of stress, injury, infection, immune response and metabolic disorders (6). Aging is one of the most prominent risk factors for various malignancies (7). There is substantial evidence of a bidirectional relationship between aging and malignant diseases, both of which share numerous common characteristics (8). Notably, cellular aging processes contribute to the pathogenesis of MM (9). Additionally, aging-related dysfunction of T cells and the immune system can exacerbate MM onset and progression (10–12). Thus, investigating aging at the molecular level may offer novel insights for the clinical translation of MM therapies.

By integrating bulk profiles with machine learning algorithms [least absolute shrinkage and selection operator (LASSO) regression and support vector machine recursive feature elimination (SVM-RFE)], this study identified TXN as an aging-associated diagnostic biomarker for MM. TXN functions as a pro-inflammatory factor by activating the NF-κB signaling pathway and generating reactive oxygen species (ROS) (13,14). *In vitro* assays further confirmed its upregulated expression in MM cell lines, highlighting its pathogenic potential.

## Materials and methods

### ARGs and sequencing dataset

In this study, 307 genes were selected from the Human Ageing Genomic Resources (http://hagr.ageing-map.org/). The mRNA expression profile datasets GSE6477 and GSE16558 were downloaded from the Gene Expression Omnibus database (https://www.ncbi.nlm.nih.gov/geo/). The GSE6477 dataset was derived from the GPL96 (HG-U133A) Affymetrix Human Genome U133A Array, while the GSE16558 dataset was derived from the GPL6244 (HuGene-1_0-st) Affymetrix Human Gene 1.0 ST Array. GSE6477 contains bone marrow CD138^+^ samples from 125 patients with MM and 15 normal bone marrow CD138^+^ controls, while GSE16558 includes bone marrow CD138^+^ samples from 60 patients with MM and 5 normal bone marrow CD138^+^ controls. The bulk data were preprocessed using R software (version 4.2.3), with batch effects removed using the ‘SVA’ package and normalization performed using the ‘normalize’ package for subsequent analysis. All datasets included the corresponding clinical information for both patients with MM and normal individuals.

### Screening of differentially expressed genes (DEGs)

DEGs between MM and normal bone marrow were identified in the integrated GSE6477 and GSE16558 dataset using a corrected P-value <0.05 and |log_2_fold change (FC)|>1 as screening criteria. The ‘limma’ package was used for DEG analysis and the intersection of aging-related genes (ARGs) was further identified to obtain aging-related DEGs (ARDEGs). The Wilcoxon rank-sum test was employed to analyze the expression of ARDEGs between MM and normal samples. Cluster analysis was performed on the selected DEGs, and visualizations including heatmaps, volcano plots and box plots were generated using the ‘pheatmap’ and ‘ggplot2’ packages.

### Protein-protein interactions (PPI) and correlation analysis of ARDEGs

To examine the interactions between ARDEGs, the Search Tool for the Retrieval of Interacting Genes and proteins (STRING) database (https://string-db.org/) was utilized. The interaction network was constructed with a confidence score threshold of >0.7. The PPI network was visualized and further analyzed using Cytoscape software (version 3.8.1; http://cytoscape.org/). The Pearson correlation was calculated using the ‘correlation graph’ function in R software to identify the relationships between ARDEGs.

### Gene Ontology (GO) and pathway enrichment analysis of ARDEGs

GO, Kyoto Encyclopedia of Genes and Genomes (KEGG) and Disease Ontology (DO) enrichment analyses were performed using the ‘GOplot’, ‘KEGGplot’ and ‘DOplot’ packages in R software. GO analysis was performed for the categories biological process, cellular component and molecular function, while KEGG analysis examined the signaling pathways of ARDEGs. DO analysis was used to explore disease enrichment. A P<0.05 was considered indicative of significantly differential enrichment of DEGs.

### Machine learning screening for ARGs

LASSO regression analysis of ARDEGs was conducted using the ‘glmnet’ package on the integrated GSE6477 and GSE16558 datasets. The SVM-RFE algorithm was applied using the ‘e1071’ package and the ‘caret’ package was used to intersect the results of the two algorithms to identify ARGs in MM.

### Evaluating the potential value of candidate genes

To validate the potential diagnostic value of the candidate genes, receiver operating characteristic (ROC) curves were plotted and the area under the ROC curve (AUC) was calculated using the ‘pROC’ package.

### Validation of ARGs in other datasets

The GSE39754 and GSE5900 datasets were used to validate the expression patterns of the candidate genes. GSE39754 was derived from the GPL5175 (HuEx-1_0-st) Affymetrix Human Exon 1.0 ST Array, while GSE5900 was derived from the GPL570 (HG-U133_Plus_2) Affymetrix Human Genome U133 Plus 2.0 Array. Both datasets included bone marrow CD138^+^ samples from 170 patients with MM and 28 normal bone marrow CD138^+^ controls, providing a basis for evaluating the diagnostic efficacy of the candidate genes. Clinical information for both patients with MM and normal individuals was included in all datasets.

### Cell lines and culture

The cell lines GM12878 (human normal B lymphocyte cell line), U266 (human MM lymphocyte-like cell line) and RPMI8226 (human MM peripheral blood B lymphocyte cell line) were obtained from the Shanghai Academy of Biological Sciences. U266 and GM12878 were designated as the MM group and GM12878 was designated as the control group. All cell lines underwent short tandem repeat authentication and mycoplasma testing, conducted by the Shanghai Academy of Biological Sciences. These cell lines were cultured in complete RPMI 1640 medium supplemented with 1% penicillin-streptomycin (Gibco; Thermo Fisher Scientific, Inc.) and 10% fetal bovine serum (FBS Gibco; Thermo Fisher Scientific, Inc.). Cell incubations were performed under strictly regulated conditions of 37°C and 5% CO_2_ in a humidified incubator.

### RNA extraction and reverse transcription-quantitative polymerase chain reaction (RT-qPCR)

Total RNA was extracted using TRIzol reagent (Takara Bio, Inc.), and its concentration, purity and integrity were evaluated with a NanoDrop™2000 spectrophotometer (Thermo Fisher Scientific, Inc.). According to the manufacturer's instructions, RT was performed using 1 µg of total RNA, processed with HiScript II Q RT SuperMix for qPCR (+gDNA wiper) (Vazyme Biotech Co., Ltd.) along with a gDNA eraser (Vazyme Biotech Co., Ltd.). The concentration, purity and integrity of the resulting cDNA were subsequently assessed using the same NanoDrop spectrophotometer. Real-time qPCR was carried out using SYBR Green MasterMix (cat. no. 11203ES50; Yeasen Biotechnology Co., Ltd.) and StepOne Software v.2.3 (Applied Biosystems; Thermo Fisher Scientific, Inc.).

The reaction conditions were as follows: Pre-denaturation at 95°C for 5 min, followed by 40 cycles of denaturation at 95°C for 30 sec, annealing at 55°C for 30 sec and extension at 72°C for 30 sec, with a final extension at 72°C for 10 min, with three biological replicates for each sample. Data analysis was conducted using the 2^−∆∆Cq^ method (15), normalizing against the expression levels of the reference gene GAPDH. The primer sequences used in the RT-qPCR assays are provided in Table I.

### Statistical analysis

All statistical analyses in this bioinformatics study were performed using R software (version 4.2.2). Pearson correlation analysis was conducted to explore associations between different variables. A P-value or false discovery rate threshold of <0.05 was set as the criterion for statistical significance. For the experimental component, statistical analyses were conducted using GraphPad Prism (version 8.0.2; Dotmatics), with each experiment including at least three biological replicates. Values are expressed as the mean ± SD. Differences between the two datasets were evaluated using either two-way ANOVA (no post-hoc test was performed) or Student's t-test, with P<0.05 considered statistically significant.

## Results

### Identification of ARDEGs based on MM-retrospective analysis

**Figure 1.**
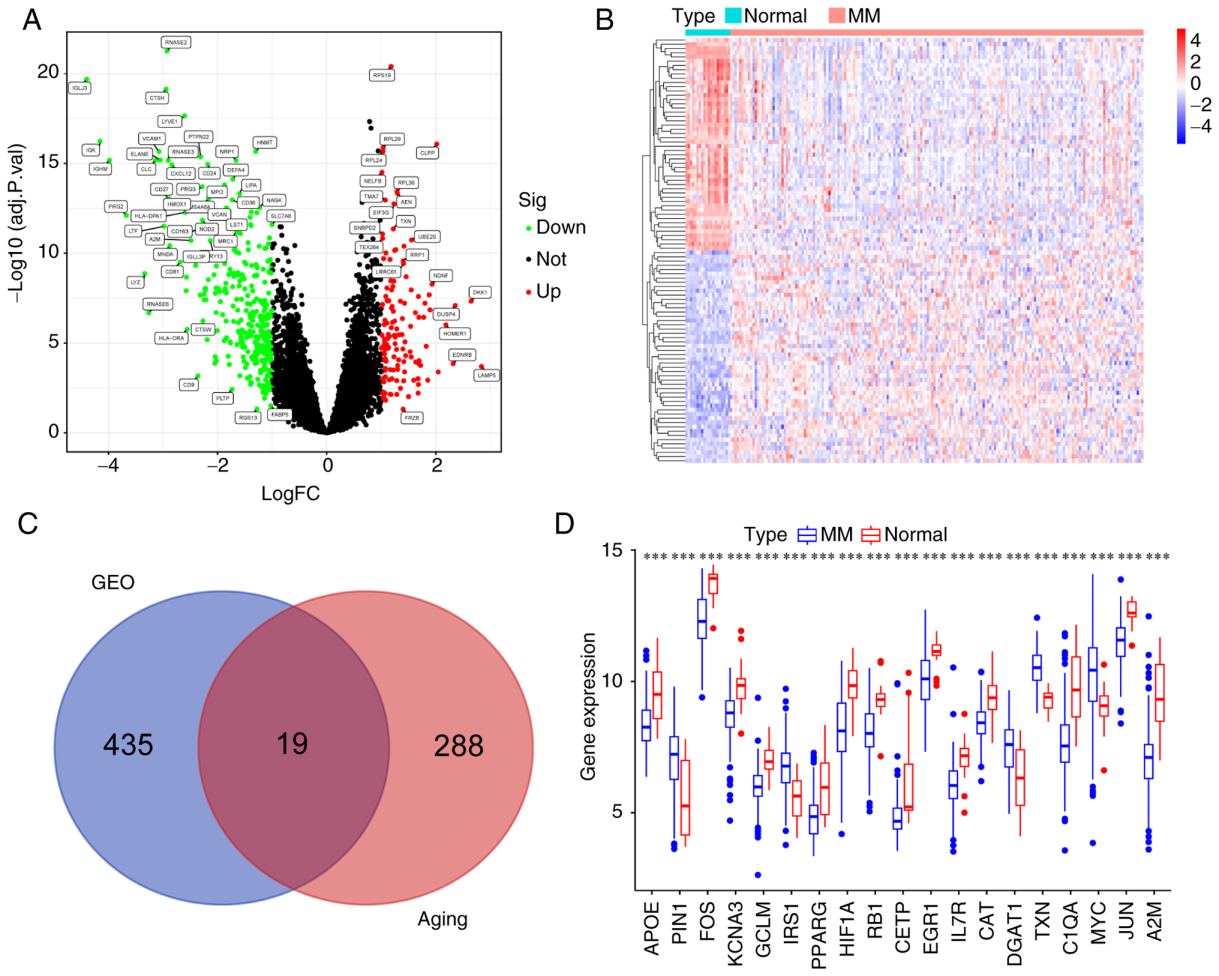
ARGs in MM and normal samples. (A) Volcano plot of the 454 DEGs in MM samples. Red dots indicate significantly upregulated genes, while green dots indicate significantly downregulated genes. (B) Heatmap of the 454 DEGs in MM samples. Colors in the heat map indicate the fold changes of differential expression (C) Venn diagram showing the identification of ARGs in patients with MM. (D) Box plot of the 19 ARGs in MM and normal samples. ***P<0.001. DEG, differentially expressed gene; ARGs, aging-related DEGs; adj.P.Val, adjusted P-value; MM, multiple myeloma; GEO, gene expression omnibus.

After preprocessing the integrated GSE6477 and GSE16558 dataset, differential gene analysis was conducted on bone marrow CD138^+^ samples from 185 patients with MM and 20 normal CD138^+^ samples using the merged data (from GSE6477 and GSE16558). A correction with criteria of P<0.05 and |log_2_FC|>1 yielded 454 DEGs, which are depicted in the volcano plot and heatmap (Fig. 1A and B). Additionally, principal component analysis (PCA) was performed to assess the overall variance and clustering of samples, as shown in Fig. S1. By intersecting these DEGs with the Aging-Associated Gene Library, 19 ARGs linked to MM were identified, including A2M, APOE, PIN1, FOS, KCNA3, GCLM, IRS1, PPARG, HIF1A, RB1, CETP, EGR1, IL7R, CAT, DGAT1, TXN, C1QA, MYC and JUN (Fig. 1C). Among these, 5 genes were upregulated, while 14 genes were downregulated in patients with MM compared to normal controls. The box plot illustrates significant expression differences of these 19 MM-related aging genes between MM and normal samples (Fig. 1D). PIN1, TXN and MYC were the top three upregulated genes, whereas A2M, C1QA and HIF1A were the top three downregulated genes (Table II).

### PPI network and correlation analysis of ARDEGs

**Figure 2.**
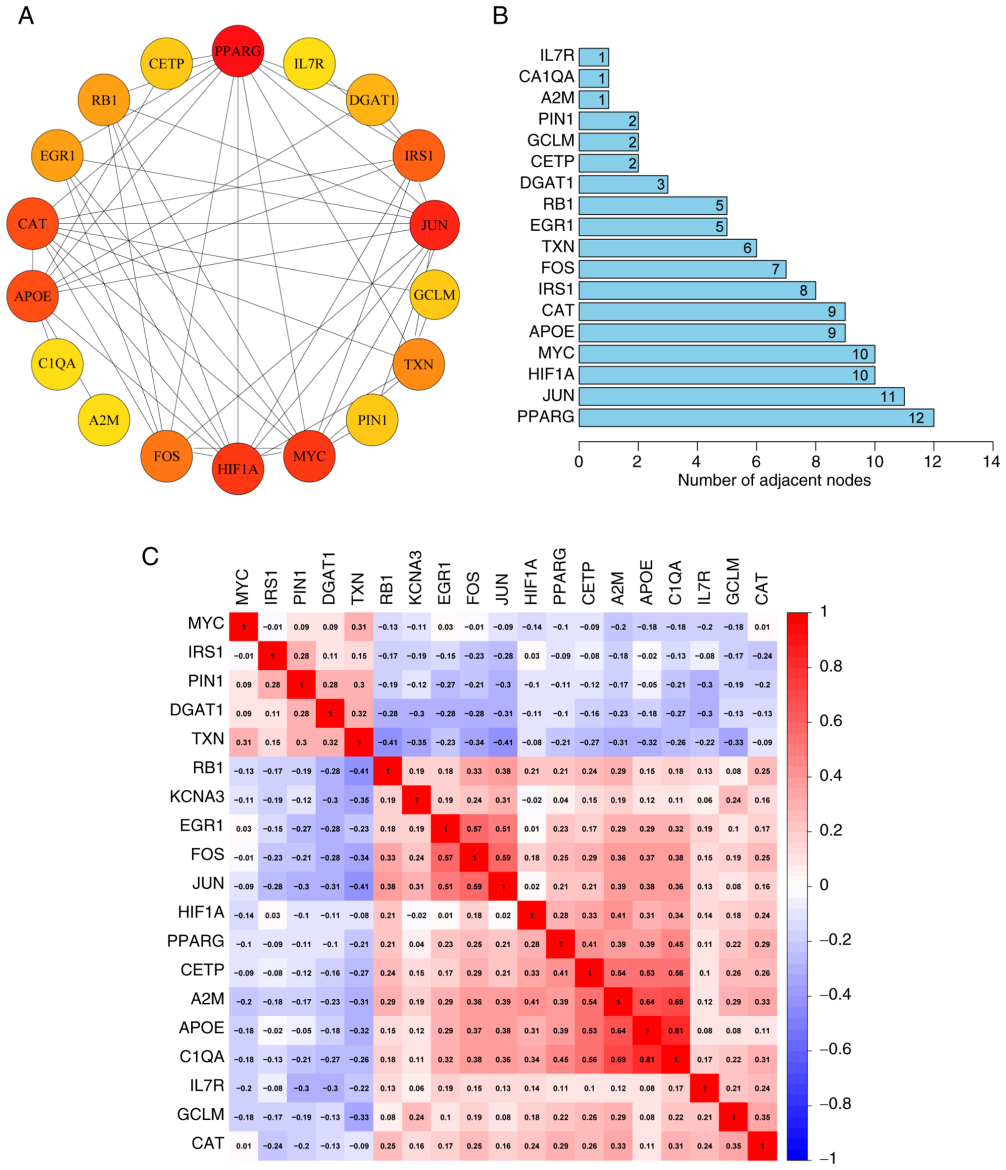
PPI analysis of the 19 ARGs. (A) PPIs among the 18 ARGs. (B) Interaction count for each ARG. (C) Pearson correlation analysis of the 18 ARGs. Numbers and related colors in the figure indicate the Pearson correlation coefficient. PPI, protein-protein interaction; ARGs, aging-related differentially expressed genes.

PPI analysis was performed to examine the interactions among the 19 ARDEGs (Fig. 2A), and the number of genes interacting with each other was quantified (Fig. 2B). The analysis revealed that 18 genes interacted with at least one other gene. PPARG interacted with 12 genes, JUN with 11, and both MYC and HIF1A interacted with 10 genes. Correlation analysis of the expression levels of the 18 interacting genes showed significant associations between them. Notably, the expression levels of C1QA, APOE and A2M were significantly positively correlated (r>0.6). The top three genes associated with C1QA were APOE (r=0.81), A2M (r=0.69) and CETP (r=0.56). For TXN, the top three associated genes were JUN (r=−0.41), RB1 (r=−0.41) and KCNA3 (r=−0.35) (Fig. 2C).

### Enrichment analysis of ARDEGs

**Figure 3.**
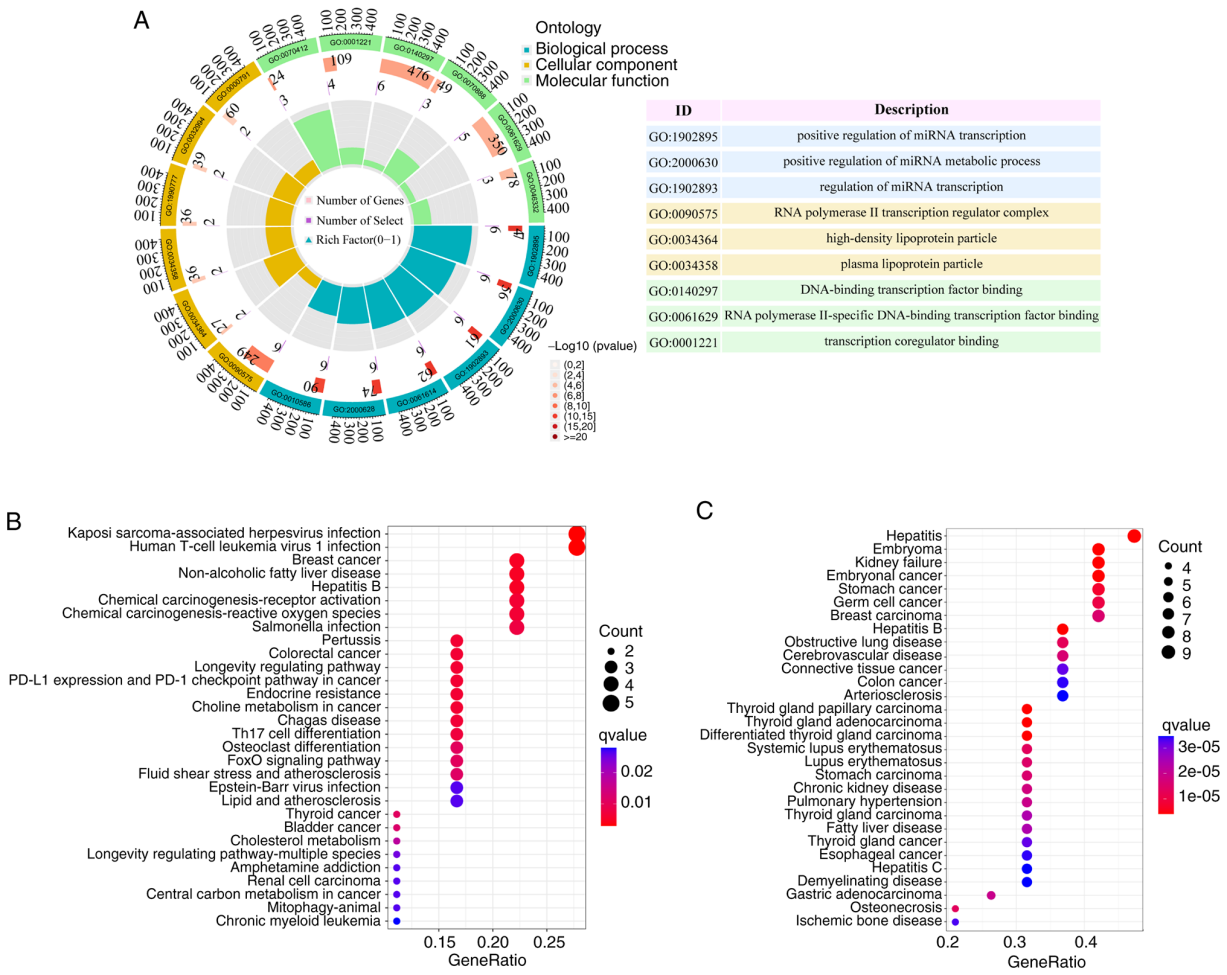
Enrichment analysis of ARGs. (A) GO enrichment analysis of 19 ARGs. (B) Kyoto encyclopedia of genes and genomes enrichment analysis of 19 ARGs. (C) DO enrichment analysis of 19 ARGs. ARGs, aging-related differentially expressed genes; GO, gene ontology; DO, disease ontology.

GO, KEGG and DO enrichment analyses were conducted using R software to determine the potential biological functions of the ARDEGs. The most significant GO enrichment terms included the ‘positive regulation of miRNA transcription’, ‘regulation of miRNA transcription’ (biological process), ‘RNA polymerase II transcription regulator complex’, ‘high-density lipoprotein particle’, ‘plasma lipoprotein particle’ (cellular component), ‘DNA-binding transcription factor binding’, ‘RNA polymerase II-specific DNA-binding transcription factor binding’ and ‘transcription co-regulator binding’ (molecular function) (Fig. 3A). KEGG enrichment analysis revealed that the ARDEGs were primarily involved in signaling pathways related to Kaposi sarcoma-associated herpesvirus infection and human T-cell leukemia virus 1 infection, suggesting their role in the dynamic regulation of the immune system (Fig. 3B). DO enrichment analysis showed that ARDEGs are crucial in the occurrence and development of hepatitis (Fig. 3C).

### Screened candidate genes of MM

**Figure 4.**
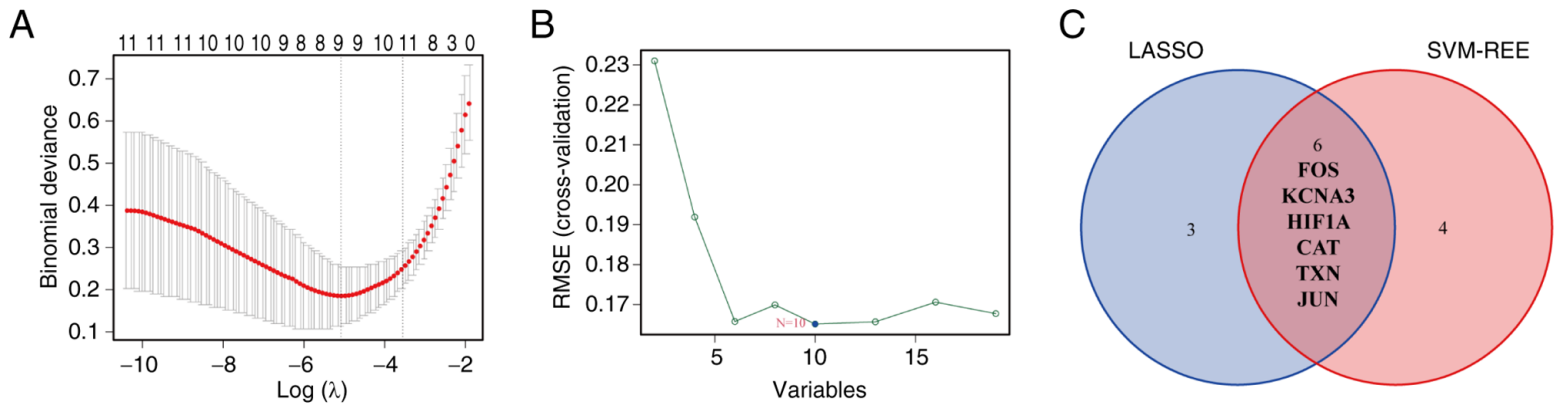
Screening of candidate genes. (A) LASSO logistic regression algorithm for candidate gene selection. (B) SVM-RFE algorithm for candidate gene selection. (C) Venn diagram showing the intersection of diagnostic markers identified by the two algorithms. LASSO, least absolute shrinkage and selection operator; SVM-RFE, support vector machine recursive feature elimination; RMSE, root mean square error, a measure of the differences between values predicted by a model and the actual observed values.

The LASSO regression algorithm identified 9 genes among the 19 MM ARDEGs: APOE, FOS, KCNA3, IRS1, PPARG, HIF1A, CAT, TXN and JUN (Fig. 4A). In comparison, the SVM-RFE algorithm identified 10 genes: TXN, RB1, A2M, FOS, JUN, GCLM, CAT, KCNA3, HIF1A and IL7R (Fig. 4B). A total of six candidate genes, overlapping between the two algorithms, were selected: FOS, KCNA3, HIF1A, CAT, TXN and JUN (Fig. 4C). Notably, all these 6 molecules are closely associated with aging.

### ROC curves of six candidate genes in MM and normal bone marrow

**Figure 5.**
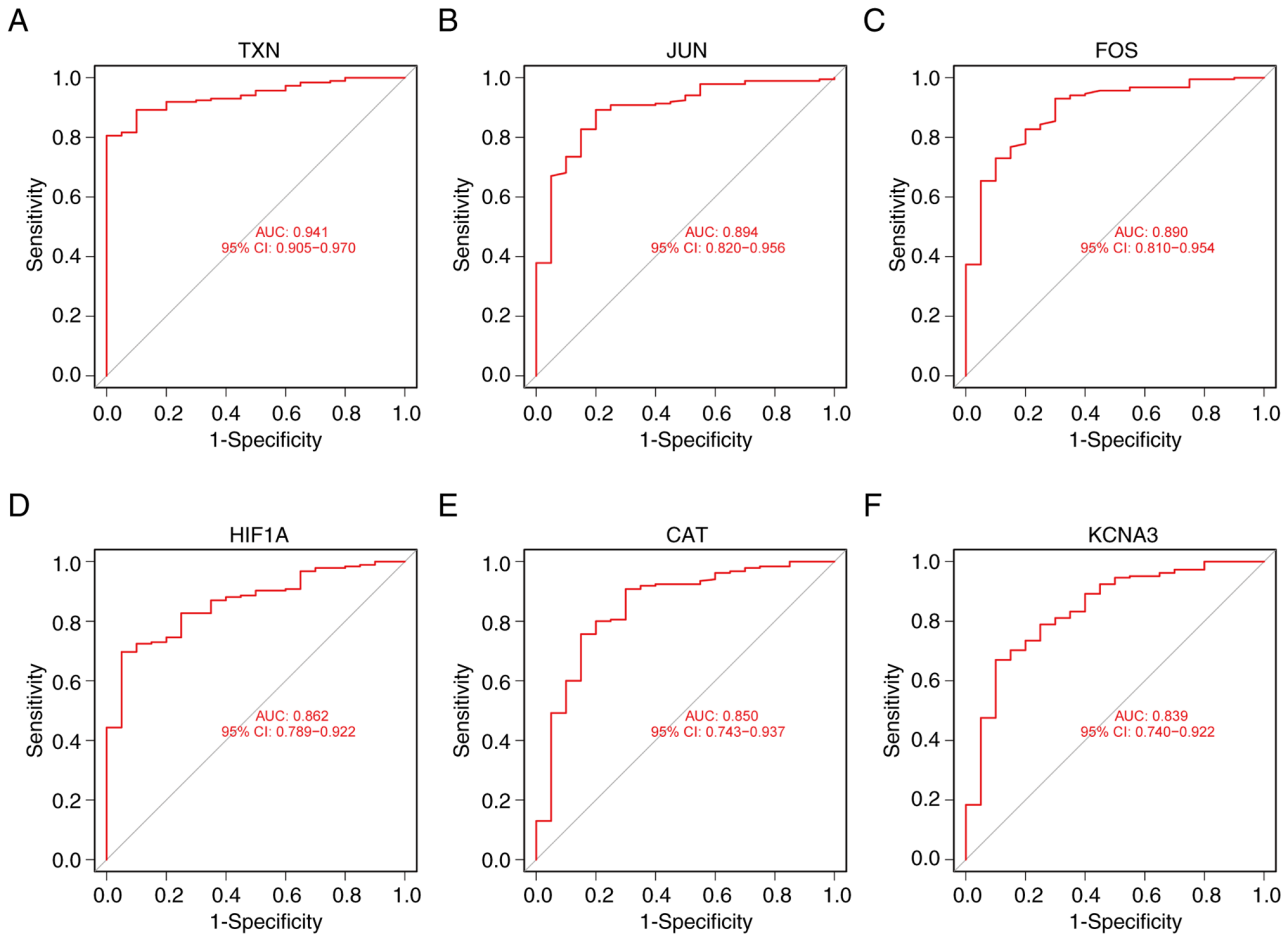
ROC curves for the diagnostic performance of the six candidate genes for multiple myeloma vs. normal samples. (A) TXN; (B) JUN; (C) FOS; (D) HIF1A; (E) CAT; and (F) KCNA3. AUC, area under the ROC curve; ROC, receiver operating characteristic.

The ‘pROC’ package was used to analyze the expression of six candidate genes in 185 MM and 20 normal bone marrow samples from the GSE6477 and GSE16558 datasets, generating ROC curves. The AUC, which combines sensitivity and specificity, was used to assess the diagnostic effectiveness of these genes. The six candidate genes demonstrated high diagnostic value for MM. Among them, TXN exhibited the highest diagnostic value in MM samples (AUC=0.941). The diagnostic values for the other genes were as follows: JUN (AUC=0.894), FOS (AUC=0.890), HIF1A (AUC=0.862), CAT (AUC=0.850) and KCNA3 (AUC=0.839) (Fig. 5A-F).

### Validation of candidate genes in other datasets

**Figure 6.**
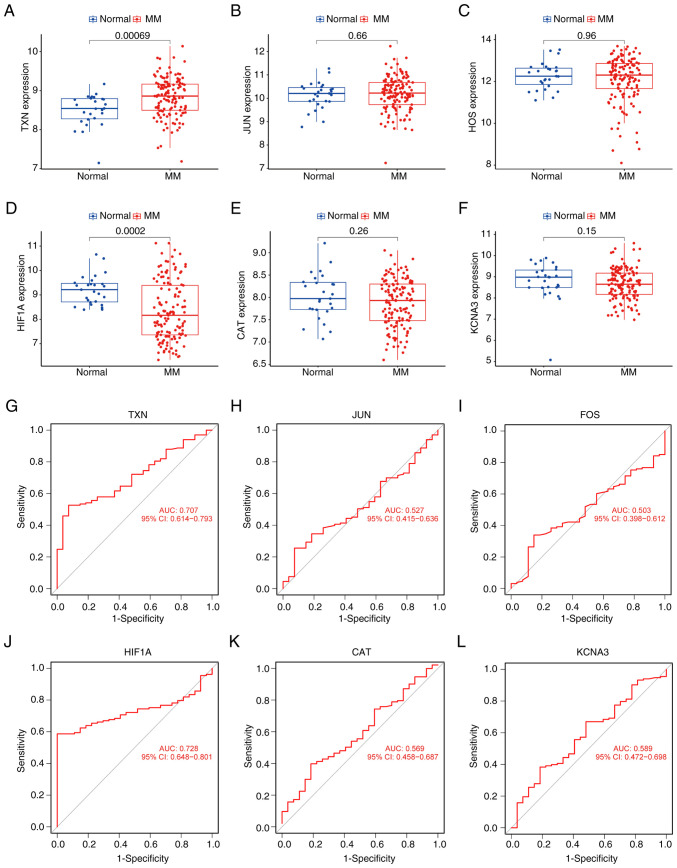
Validation of hub genes in other datasets. (A-F) Expression levels of the candidate diagnostic markers (A) TXN, (B) JUN, (C) FOS, (D) HIF1A, (E) CAT and (F) KCNA3 in the MM and normal samples in the validation cohort. (G-L) ROC curve of the six candidate genes in MM and normal samples (validation dataset). (G) TXN, (H) JUN, (I) FOS, (J) HIF1A, (K) CAT and (L) KCNA3. AUC, area under the ROC curve; ROC, receiver operating characteristic; MM, multiple myeloma.

The GSE39754 and GSE5900 datasets were used as a validation cohort to assess the accuracy of the analysis results and the expression levels of the six candidate genes. As shown in Fig. 6A-F, a significant difference in TXN and HIF1A expression was observed between MM and normal samples. TXN was overexpressed and HIF1A expression was reduced in MM samples. Notably, HIF1A expression was more variable in the MM samples. Additionally, the ROC curve analysis in the validation cohort (Fig. 6G-L) showed that TXN (AUC=0.707), JUN (AUC=0.527), FOS (AUC=0.503), HIF1A (AUC=0.728), CAT (AUC=0.569) and KCNA3 (AUC=0.589) exhibited diagnostic value. Among these, TXN had the highest diagnostic value and can serve as a key diagnostic marker for MM.

### Verification of the key genes in the human MM cell line

**Figure 7.**
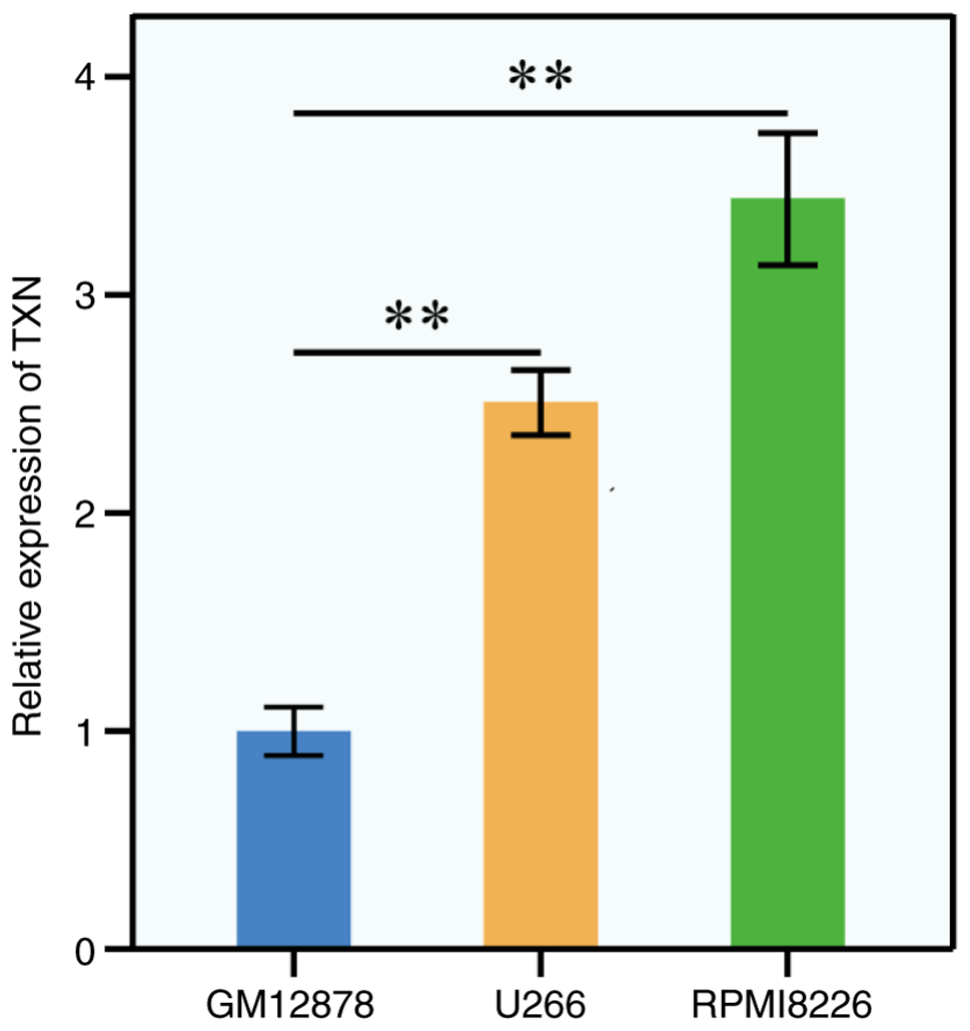
Relative mRNA expression levels of TXN analyzed in in normal and multiple myeloma cell lines. GM12878, a normal, non-cancerous lymphoblastoid cell line, was set to 1 for normalization and used as the reference for comparison with the U266 and RPMI8226 cancer cell lines. Error bars represent the standard deviation (or standard error of the mean) from at least three independent experiments. **P<0.01 compared with GM12878.

To validate the bioinformatics results, RT-qPCR was performed on the human MM cell lines U266 and RPMI8226 to further analyze TXN expression. The results from the U266 and RPMI8226 cell validation tests were consistent with the bioinformatics analysis, showing significantly higher expression levels of TXN compared to normal cells. Specifically, the expression of TXN was significantly greater in U266 (P<0.01) and RPMI8226 (P<0.01) cells than in normal cells (Fig. 7).

## Discussion

In the present study, bioinformatics analysis and machine learning techniques were utilized to identify aging-related diagnostic and druggable targets for patients with MM. The results demonstrated that TXN could serve as a robust diagnostic biomarker for detecting MM pathogenesis. Additionally, the potential biological and molecular functions of TXN in MM were explored, offering novel insights into the disease's underlying mechanisms.

Aging is the primary risk factor for most malignancies (5). For instance, MM is an aging-related disease that poses significant health challenges for the elderly (16). Epidemiological investigation pointed out the global burden of MM is increasing in numerous countries due to an aging population (17). Studies have highlighted aging-associated epigenetic and genetic alterations that characterize both MM development and normal aging processes (9). For example, aging can destroy the immune systems, which leads to the impairment of immune surveillance and pathogenesis of MM (18). Besides, enhancer of zeste homolog 2 inhibition induces senescence via the ERK1/2 signaling pathway in MM (19). Additionally, inositol-requiring enzyme 1α inhibition enhances mitochondrial quality in CD8^+^ T cells, thereby improving their anti-tumor efficacy (20). Furthermore, genetic instability in patients with MM contributes to an anti-senescence effect, promoting disease progression (21). Age-related mesenchymal stromal cell senescence has also been linked to the progression from monoclonal gammopathy of undetermined significance to MM (22). In the present study, 6 molecules were identified (FOS, KCNA3, HIF1A, CAT, TXN and JUN) that are closely associated with aging. The m6A mRNA demethylase FTO in granulosa cells can slow FOS-dependent ovarian aging (23). KCNA3 is implicated in cognitive aging (24). P53/HIF-1α regulates neuronal aging and autophagy in spinal cord ischemia/reperfusion injury (25). CAT contributes to autophagy-induced vascular smooth muscle cell senescence (26). Additionally, Astragalus polysaccharide alleviates oxidative stress and senescence in osteoarthritis chondrocytes *via* the GCN2/ATF4/TXN axis (27). Activation of JUN has long been considered a hallmark of cellular senescence (28). The present study confirmed that TXN can serve as a promising diagnostic biomarker for MM, a malignant blood cancer characterized by uncontrolled plasma cell proliferation. TXN plays a pivotal role in the pathophysiology of MM, affecting both the cellular aging process and the maintenance of cellular redox balance. This study further explored the novel mechanisms through which TXN contributes to MM, providing valuable insights into the disease's pathophysiology. Additionally, the TXN-ALDH1L2 axis has been shown to promote the progression of colorectal cancer and radioresistance by activating the NF-κB signaling pathway (14), which is also closely associated with MM pathogenesis (29). Thus, TXN may regulate NF-κB signaling to modulate MM progression.

Despite these insights, there are still gaps in our understanding. Specifically, further studies are needed to elucidate the precise mechanisms by which TXN acts in MM, as well as to develop targeted therapeutic strategies based on TXN's regulatory pathways, particularly regarding its role in apoptosis regulation. Furthermore, additional research is required to identify therapeutic agents targeting TXN for MM treatment, which could enhance the clinical applicability of the present findings. The *in vitro* results obtained from the GM12878 EBV-transformed lymphoblastoid cell line should also be validated in patient samples to ensure authenticity. Besides, ARGs identified in the present study that are also involved in the pathogenesis of other disease or biological functions showed a weaker association with MM pathogenesis, indicating a need for further pre-clinical validation of these DEG mechanisms in MM. Furthermore, differences in the diagnostic performance of TXN between the training and validation sets of patients with MM suggest predictive heterogeneity. Therefore, future studies should assess TXN's diagnostic performance in multi-center cohorts to strengthen its diagnostic robustness. Lastly, the impact of TXN dysregulation on the overall survival of patients with MM should be explored in clinical studies to provide further evidence for the clinical relevance of TXN.

## Supplementary Material

Supporting Data

## Figures and Tables

**Table I.** PCR primers used in this study.

TXN forward primer	5′-CTGGATTATGCAGAGTACGTTCG-3′
TXN reverse primer	5′-CGACTTGCTGCTTGCTCAATTT-3′
GADPH forward primer	5′-TGACTTCAACAGCGACACCCA-3′
GADPH reverse primer	5′-CACCCTGTTGCTGTAGCCAAA-3′

**Table II.** Comparison of the 19 ARDEGs in multiple myeloma samples with those in normal samples.

Gene symbol	Log_2_FC	Direction of differential expression	P-value	Adjusted P-value	Chromosome
PIN1	1.413596257	Up	9.50×10^−6^	1.08×10^−4^	19p13
TXN	1.221137514	Up	2.28×10^−14^	4.40×10^−12^	9q31.3
MYC	1.177462797	Up	1.59×10^−3^	7.38×10^−3^	8q24.21
IRS1	1.100398111	Up	7.24×10^−7^	1.23×10^−5^	2q36.3
DGAT1	1.079197683	Up	8.10×10^−6^	9.50×10^−5^	8q24.3
A2M	−2.48525999	Down	1.20×10^−13^	1.94×10^−11^	12p13.31
C1QA	−2.027119477	Down	1.47×10^−10^	8.60×10^−9^	1p36.12
HIF1A	−1.61783154	Down	3.65×10^−8^	7.35×10^−7^	14q23.2
FOS	−1.473078631	Down	4.39×10^−9^	1.54×10^−7^	14q24.3
CETP	−1.353050943	Down	5.50×10^−9^	1.90×10^−7^	16q13
RB1	−1.284638924	Down	4.99×10^−9^	7.73×10^−7^	13q14.2
PPARG	−1.22131096	Down	1.04×10^−8^	3.30×10^−7^	3p25.2
APOE	−1.198617718	Down	5.17×10^−8^	1.29×10^−6^	19q13.32
KCNA3	−1.196436349	Down	1.88×10^−7^	6.92×10^−6^	1p13.3
JUN	−1.146427429	Down	1.08×10^−8^	3.40×10^−7^	1p32.1
EGR1	−1.033395792	Down	3.45×10^−5^	3.23×10^−4^	5q31.2
GCLM	−1.006391329	Down	4.29×10^−9^	1.52×10^−7^	1p22.1
IL7R	−1.001299766	Down	2.55×10^−6^	3.60×10^−5^	5p13.2
CAT	−1.000447561	Down	1.12×10^−9^	4.82×10^−8^	11p13

The ‘limma’ package was used for DEG analysis (P-value <0.05 and |log_2_FC| >1 as screening criteria) and the intersection of aging-related genes was further identified to obtain ARDEGs. FC, fold change; ARDEGs, aging-related differentially expressed genes.

## Data Availability

The data generated in the present study may be requested from the corresponding author.
