# Supplementary material for: Identification and validation of aging-related genes in patients with multiple myeloma
Source: Oncol Lett. 2026 Jul 2;32(3):384. doi: 10.3892/ol.2026.15739 (PMC13351648; doi:10.3892/ol.2026.15739)

Figure S1. Pre-processing results of integrated GSE6477 and GSE16558 datasets. PC analysis was performed using normalized gene expression data to visualize sample similarity and variance. PC1 and PC2 represent the top two dimensions that explain the greatest proportion of overall variation in the dataset. MM, multiple myeloma; PC, principal component.

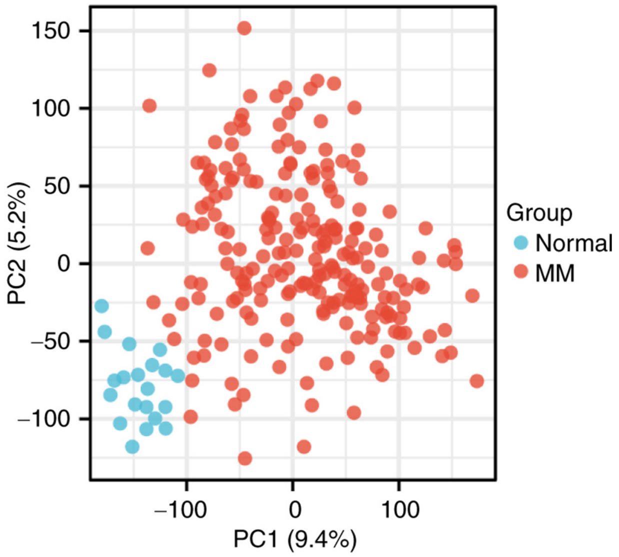

Supplement: Supporting Data [file Supplementary_Data.pdf]
